# The effect of a modified Peyton's four-step teaching method on orthokeratology skills: an experimental study

**DOI:** 10.3389/fmed.2026.1791647

**Published:** 2026-05-11

**Authors:** Jing Wang, Ke Zhang, Qianqian Wan, Yuwei Zheng, Liming Tao, Lin Ling

**Affiliations:** 1 Department of Ophthalmology, The Second Affiliated Hospital of Anhui Medical University, Hefei, China; 2 Department of Obstetrics and Gynecology, The Second Affiliated Hospital of Anhui Medical University, Hefei, China

**Keywords:** medical education, ophthalmology, optometry, orthokeratology, Peyton's four-step teaching method

## Abstract

**Purpose:**

This study aimed to evaluate the effectiveness of a modified Peyton's four-step method in teaching orthokeratology skills.

**Methods:**

A total of 60 fourth- and fifth-year optometry students undertaking internships at the Optometry Center of the Second Affiliated Hospital of Anhui Medical University in December 2025 were enrolled. The students were randomly assigned to an experimental group or a control group, with 30 students in each group. The experimental group received training in basic orthokeratology skills using the modified Peyton's four-step teaching method, whereas the control group was trained using the traditional “See One, Do One” approach. The total training duration was four credit hours. One week after completion of the training, skill-based operational assessments and teaching satisfaction questionnaires were administered to both groups. Statistical analyses were conducted using the *t*-test and the chi-square test.

**Results:**

For lens cleaning, lens wearing, trial wear evaluation, and total scores, the experimental group achieved significantly higher scores than the control group (*P* < 0.05). No statistically significant difference was observed between the two groups in scores for determining lens parameters (*P* > 0.05). The satisfaction questionnaire showed that students in the experimental group reported significantly higher scores for teaching arrangement, teaching methods, interactive feedback and participation compared with the control group (*P* < 0.05). There were no statistically significant differences between the two groups in scores for assessment methods or overall satisfaction with teaching (*P* > 0.05).

**Conclusion:**

The modified Peyton's four-step teaching method demonstrated favorable outcomes in training basic orthokeratology skills and achieved high trainee satisfaction, indicating that it is worthy of wider adoption.

## 1 Introduction

Orthokeratology is an important component of myopia prevention and control strategies for adolescents and represents a fundamental skill that optometry students are required to master. However, teaching in this area currently faces several challenges, including extensive course content, limited class hours, numerous operational steps, and difficulties in memorization. The traditional approach to teaching orthokeratology skills is predominantly the “See One, Do One” method, first proposed by Dr Halsted in 1904. This approach is based on a two-step learning model of “observation–imitation” (1). Owing to insufficient supervision and reflection on performance, the “See One, Do One” model has limitations in enhancing students' cognitive abilities (2, 3), professional knowledge, and in shortening the learning curve (4, 5). Based on his experience and observations in surgical resident training, Dr Peyton proposed a clinical skills teaching approach in 1998, known as Peyton's Four-Step Teaching Method, which comprises demonstration, deconstruction, comprehension, and practice (6). Previous studies have shown that Peyton's Four-Step Teaching Method can improve medical students' skills in tracheal intubation (7) and laparoscopic procedures (8). However, evaluations of its effectiveness in teaching musculoskeletal and cardiac ultrasound have not demonstrated significant advantages over traditional teaching methods (9, 10). The classic Peyton's Four-Step Teaching Method was originally designed for teaching contexts with a teacher–student ratio of 1:1. In routine medical education, however, it is more common for one instructor to teach multiple students simultaneously. To address this issue, medical education experts developed a modified Peyton teaching method comprising six steps, enabling all students to actively participate in the teaching process (11). This modified approach has been applied to training in spinal manipulation (12) and physical diagnostics (13), with findings indicating that the modified Peyton's teaching method is an effective strategy for teaching complex operational skills. Therefore, orthokeratology lens fitting involves a highly sensitive anatomical site, requires stringent precision, and relies heavily on patient cooperation. Consequently, it imposes higher demands on medical students in terms of both doctor-patient communication skills and operational proficiency.the present study aimed to investigate the application of the modified Peyton's four-step teaching method in orthokeratology training for optometry students.

## 2 Materials and methods

### 2.1 General information

A total of 60 fourth- and fifth-year optometry students undertaking internships at the Optometry Center of the Second Affiliated Hospital of Anhui Medical University in December 2025 were enrolled in this study. The students were randomly assigned to an experimental group or a control group using the lottery method, with 30 students in each group. Fourth-students had completed all optometry courses, and the two groups had equivalent levels of experience with orthokeratology, and all participants were receiving training in orthokeratology fitting for the first time. The experimental group comprised 14 fourth-year and 16 fifth-year optometry students, and the control group consisted of 17 fourth-year and 13 fifth-year students. During the training and assessment processes, blinding was implemented, and all students were unaware of their group allocation to minimize subjective bias. Written informed consent was obtained from all participants. The study was approved by the Ethics Committee of the Second Affiliated Hospital of Anhui Medical University (YX2026-007). The experimental group received training in basic orthokeratology skills using the modified Peyton's four-step teaching method, whereas the control group was trained using the traditional “See One, Do One” approach. Both groups completed a single training session comprising a total of four contact hours. The training content included cleaning of orthokeratology lenses, lens wearing, trial wear evaluation, and determination of lens parameters. A unified assessment was conducted one week after completion of the course.

### 2.2 Research methods

#### 2.2.1 Modified Peyton's teaching method

**Figure 1:**
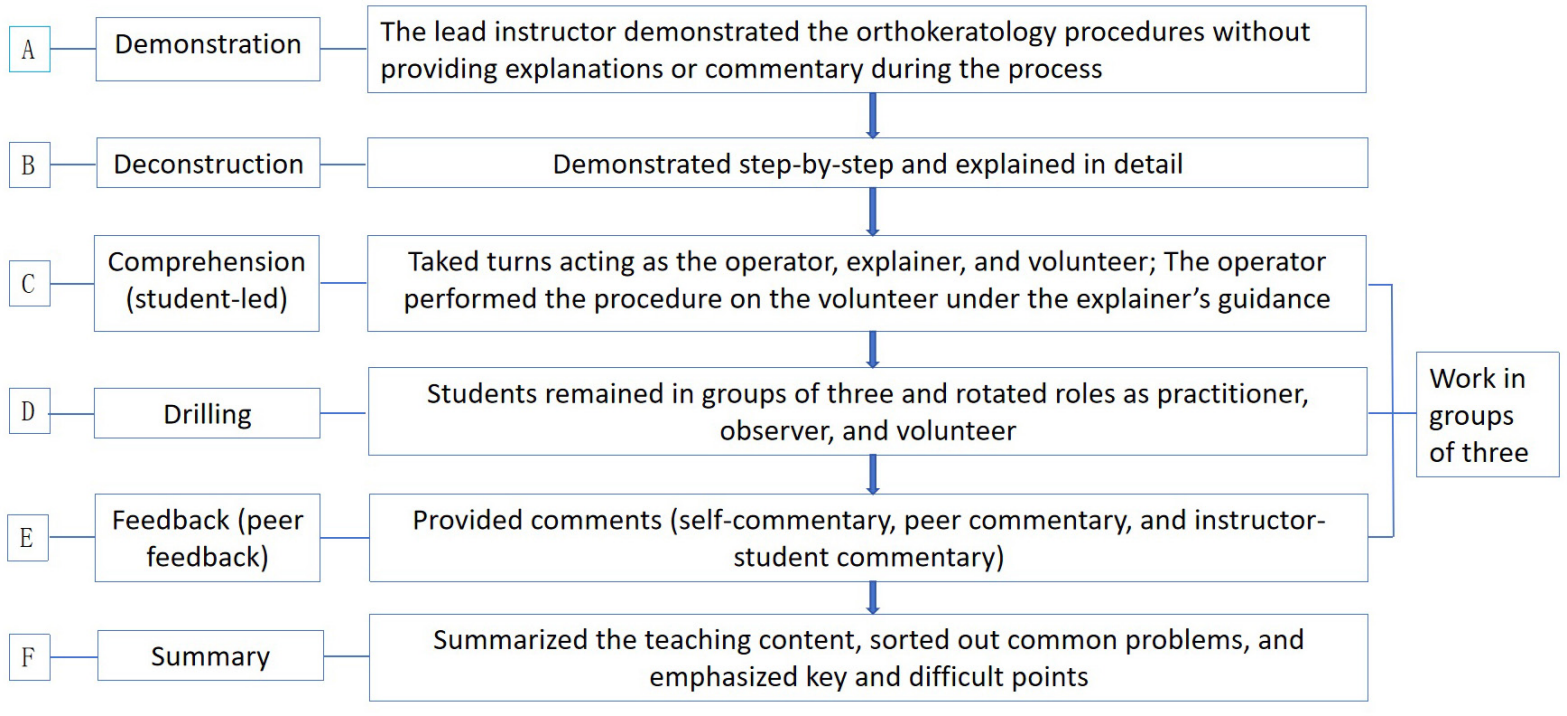
Modified Peyton's four-step teaching method.

The research team modified the classic Peyton teaching method by retaining its core instructional steps and incorporating the concept of “peer learning”. The implementation process was as follows. A. Demonstration: the lead instructor demonstrated the orthokeratology procedures without providing explanations or commentary during the process. B. Deconstruction: the instructor demonstrated each step sequentially and provided detailed explanations, requiring students to learn and record each operational sub-step. C. Comprehension (student-led): this stage represented a key distinction from traditional teaching methods. Students were divided into groups of three and took turns acting as the operator, explainer, and volunteer. The explainer described the procedure according to the sub-steps taught by the instructor, observed the operator's performance, and explained the function and significance of each sub-step. The operator performed the procedure on the volunteer under the explainer's guidance. The instructor observed and provided timely correction and guidance until all students were able to understand, explain, and correctly perform each sub-step. D. Drilling: students remained in groups of three and rotated roles as practitioner, observer, and volunteer. The practitioner independently completed the basic orthokeratology skill tasks, while the observer recorded completion time and carefully monitored the procedure to provide feedback. E. Feedback (peer feedback): group members provided comments and suggestions to one another and engaged in questioning or discussion as appropriate. F. Summary: the instructor summarized the teaching content, addressed common problems, highlighted key and difficult points, and assigned post-class practice tasks. The detailed teaching process is illustrated in Figure 1. During stages C, D, and E, the three students supervised and provided feedback to one another, effectively addressing the reduced teaching efficiency associated with an insufficient teacher–student ratio in traditional teaching models.

#### 2.2.2 Teaching methods of traditional orthokeratology

The control group adopted the traditional “See One, Do One” approach for teaching orthokeratology skills. The lead instructor demonstrated the procedures in a centralized manner and explained each operational step in sequence. After students had acquired a basic understanding of the theoretical and practical components, they practiced in groups, during which the instructor provided on-site guidance and responded to students' questions.

### 2.3 Observation indicators

#### 2.3.1 Scores of basic skills performance assessment

One week after completion of the training course, a unified skills assessment was conducted. All trainees drew lots to determine their identification numbers and examination order. The assessment was invigilated by non-teaching staff who were blinded to the students' identities, group allocation, and other relevant information. The assessment comprised lens cleaning, lens wearing, trial wear evaluation, and determination of lens parameters. Each item was scored out of 25 points, giving a total score of 100 points. Two non-teaching experts independently evaluated the trainees' performance, and the mean of the two scores was taken as the final result.

#### 2.3.2 Teaching satisfaction survey

After the course, a questionnaire survey was administered to both groups to evaluate teaching satisfaction. The survey assessed satisfaction across five aspects: teaching arrangement, teaching methods, interactive feedback and participation, assessment and evaluation methods, and satisfaction with the instructors. Each item was rated using a five-point Likert scale, with the categories “very satisfied”, “satisfied”, “average”, “somewhat dissatisfied”, and “very dissatisfied”, corresponding to scores of 5, 4, 3, 2, and 1, respectively. This assessment was based on subjective self-reporting.

### 2.4 Statistical analysis

Statistical analyses were performed using SPSS version 23.0. Measurement data conforming to a normal distribution were expressed as mean ± standard deviation, and between-group comparisons were conducted using the t-test. The Mann-Whitney U test was used for comparisons of data with a non-normal distribution. A P value < 0.05 was considered statistically significant.

## 3 Result

### 3.1 Demographic characteristics of the participants

Table 1 presents the main demographic characteristics of the two groups. The mean age of participants in the experimental group was 22.10 ± 0.55 years, compared with 22.07 ± 0.58 years in the control group. The experimental group comprised 12 males and 18 females, whereas the control group included 11 males and 19 females. No significant differences were observed between the two groups in terms of age or sex distribution at baseline (*P* > 0.05).

**Table 1:** Demographic characteristics of participants.

Characteristics	Experimental group (*n* = 30)	Control group (*n* = 30)	*P*
Age (years)	22.10 ± 0.55	22.07 ± 0.58	0.813^1^
Gender (male/female)	12/18	11/19	1.000^2^

^1^The *P*-value was derived from paired samples t-test

^2^The *P*-value was calculated using the chi-square test

### 3.2 Scores of skill operation assessment

For orthokeratology lens cleaning, lens wearing, trial wear evaluation, and total score, the experimental group achieved significantly higher scores than the control group (*P* < 0.05). However, no statistically significant difference was found between the two groups in scores for determining lens parameters (*P* > 0.05). The results are summarized in Table 2.

**Table 2:** Scores of skill operation assessment.

Variable	Experimental group (*n* = 30)	Control group (*n* = 30)	*t*	*P*
	Mean ± S.D	Mean ± S.D		
Lens cleaning	21.80 ± 1.27	21.00 ± 1.20	2.693	0.012
Lens wearing	21.40 ± 1.13	19.90 ± 0.96	4.962	0.000
Trial wear evaluation	22.00 ± 1.02	19.70 ± 0.79	9.206	0.000
Lens parameters	21.10 ± 1.40	20.80 ± 1.42	1.361	0.184
Total score	86.30 ± 2.18	81.40 ± 2.14	8.307	0.000

### 3.3 Teaching satisfaction score

A total of 60 questionnaires were distributed and all were returned, yielding a response rate of 100%. Students in the experimental group reported significantly higher scores for teaching arrangement, teaching methods, interactive feedback on topics and participation compared with the control group (*P* < 0.05). No statistically significant differences were observed between the two groups in scores for assessment methods or overall satisfaction with teaching (*P* > 0.05), as shown in Table 3.

**Table 3:** Teaching satisfaction score.

Project	Very satisfied		Satisfied		General		Not very satisfied		Very dissatisfied		Median (IQR)		*Z*	*P*
	Experimental group	Control group	Experimental group	Control group	Experimental group	Control group	Experimental group	Control group	Experimental group	Control group	Experimental group	Control group		
Teaching organization	18	12	11	10	1	8	0	0	0	0	5 (4.00, 5.00)	4 (3.00,5.00)	−2.095	0.036
Teaching methods	22	10	7	15	1	5	0	0	0	0	5 (4.00, 5.00)	4 (3.00, 5.00)	−3.144	0.002
Interactive feedback and participation	15	9	12	9	3	7	0	5	0	0	4.00 (3.25, 5.00)	4.00 (3.25, 5.00)	−2.447	0.014
Assessment methods	10	11	17	16	3	3	0	0	0	0	4 (4.00, 5.00)	4 (4.00, 5.00)	−0.225	0.822
Satisfaction with teaching	16	15	12	13	1	1	1	1	0	0	5 (4.00, 5.00)	5 (4.00, 5.00)	−0.233	0.816

## 4 Discussion

Orthokeratology, as an important method for myopia control, is an essential skill for optometry students. In previous experimental courses on orthokeratology, teaching was predominantly based on the “See One, Do One” method for explanation and demonstration. This approach has several limitations. First, in traditional group-based teaching, students mainly observe the instructor's demonstration. As the number of interns in each group is relatively large and the demonstrable operational field is limited, students who move closer to observe details may obstruct others, making it difficult to ensure consistent teaching quality. Second, limited interaction during demonstrations restricts instructors' ability to actively guide students' thinking, resulting in an unclear understanding of operational steps. Consequently, this approach fails to enhance students' cognitive abilities (2, 3), professional knowledge, or to shorten the learning curve (4, 5). In recent years, the Peyton's teaching method has been increasingly adopted for teaching complex clinical skills. The classic Peyton's teaching method is designed for one-to-one instruction; however, in routine practice, teaching is more commonly conducted in small classes. To improve practicality, a modified Peyton's four-step teaching method has been proposed, comprising six steps: demonstration, deconstruction, understanding, practice, feedback, and summary. This model has demonstrated favorable training outcomes in clinical skills education in obstetrics and gynecology, dental root canal treatment, and cardiopulmonary resuscitation (14–16). In the present study, the modified Peyton's four-step teaching method was applied to orthokeratology skills training for optometry students, incorporating the concept of “peer learning”. Prior to training, students in the experimental group received the demonstration and deconstruction stages delivered by instructors in a demonstration classroom. Through multimedia presentation, each student was able to clearly and comprehensively observe the operational procedures. Particular emphasis was placed on the stages of understanding and practice. When acting as explainer, students were required to restate the key points of each operational step; when acting as volunteers, they experienced the impact of each step on patients; and when acting as observers, they evaluated the strengths and weaknesses of their peers' performance. This process further promoted mastery of each operational skill. Throughout the training, students were encouraged to raise questions, transforming passive instruction into active learning through real-time feedback and peer discussion. Peer-based interactive activities, such as recitation practice, performance demonstrations, and mutual evaluation and feedback, not only enriched emotional engagement but also enhanced awareness of individual strengths and weaknesses. These activities improved memory retention and reflective learning abilities, while fostering a collaborative learning environment based on mutual support and complementary strengths. This approach facilitates the construction of knowledge and experiential meaning, thereby positively promoting professional identity (17–19). Peer assessment and feedback are often more readily accepted, generate stronger resonance, and accelerate understanding and memory retention. Instructors play a critical role in organizing and supervising the teaching process and providing feedback, helping students establish psychological safety and engage in in-depth reflection. Ultimately, this process improves cognitive thinking patterns and future clinical behaviors, while enhancing confidence and satisfaction in real clinical practice (20).

The results of this study showed that the experimental group achieved significantly higher scores in orthokeratology lens cleaning, lens wearing, trial wear evaluation, and total score compared with the control group. These findings indicate that the modified Peyton's four-step teaching method effectively enhances students' proficiency in complex operational skills. The structured framework of the Peyton's method promotes active student participation in the learning process (21–23). By introducing peer learning and role rotation during the stages of understanding, practice, and feedback, the modified method enables students to learn from peers' errors, reduce potential mistakes, and improve academic performance and learning motivation (24, 25). Compared with the traditional “See One, Do One” approach, the Peyton's teaching method significantly improves skill performance (26). However, no statistically significant difference was observed between the two groups in scores for determination of lens parameters. This may be attributable to the greater reliance of this task on theoretical knowledge and accumulated clinical experience. Determination of orthokeratology lens parameters requires comprehensive judgement based on multiple indicators, such as corneal morphology and refractive power, and represents a higher-level cognitive decision-making ability. In contrast, the advantages of the modified Peyton's four-step teaching method are primarily reflected in the development of practical operational skills. In addition, students in both groups had completed core optometry courses and possessed a basic theoretical foundation for parameter determination, which may have further reduced between-group differences. It should also be noted that the deconstruction and understanding stages of the Peyton's method are time-consuming. In some studies, these stages have been replaced by standardized instructional videos, allowing the Peyton's method to be implemented within a similar time frame to the “See One, Do One” approach (27).

The teaching satisfaction survey demonstrated that the experimental group reported significantly higher scores for teaching arrangement, teaching methods, interactive feedback and participation compared with the control group. This finding suggests that the modified Peyton's four-step teaching method better aligns with students' learning needs. Through multi-stage interactive sessions, this approach effectively stimulates learning initiative and participation, providing students with more opportunities for expression and hands-on practice (28). Moreover, personalized feedback enhances students' perception of targeted instructional guidance and improves the overall learning experience (29). No significant differences were observed between the two groups in scores for assessment methods or satisfaction with teaching. This may be related to the use of identical assessment criteria and the same teaching staff in both groups, as well as students' recognition of the instructors' professional competence and teaching attitudes.

## 5 Limitations

This study has several limitations. The sample size was relatively small, and the study was conducted at a single center, which may limit the generalisability of the findings. In addition, the training duration was short, and no long-term follow-up was performed to evaluate sustained skill acquisition. Future studies should include larger sample sizes, adopt multi-center designs, and extend follow-up periods to further assess the long-term effectiveness of the modified Peyton's four-step teaching method. Integration of modern educational approaches, such as virtual simulation technology, may further optimize teaching strategies and enhance training outcomes.

## 6 Conclusion

The modified Peyton's four-step teaching method, through a structured instructional design and the integration of peer learning, effectively addresses the challenges of “difficulty in practical operation, slow skill acquisition, and limited application” in orthokeratology education. This method not only significantly improves students' practical skills and teaching satisfaction, but also aligns with the core principles of “student-centered and clinically oriented” modern medical education. It therefore represents a teaching approach with strong potential for wider application in optometry clinical training.

## Data Availability

The original contributions presented in the study are included in the article/supplementary material, further inquiries can be directed to the corresponding author.
